# Preschoolers’ Induction of the Concept of Material Kind to Make Predictions: The Effects of Comparison and Linguistic Labels

**DOI:** 10.3389/fpsyg.2020.531503

**Published:** 2020-11-19

**Authors:** Ilonca Hardy, Henrik Saalbach, Miriam Leuchter, Lennart Schalk

**Affiliations:** ^1^Institute of Early and Primary Education, Educational Sciences, Goethe University Frankfurt, Frankfurt, Germany; ^2^Institute of Educational Sciences, Leipzig University, Leipzig, Germany; ^3^Leipzig Research Center for Early Childhood Development, Leipzig University, Leipzig, Germany; ^4^Institute for Children and Youth Education, Educational Sciences, University of Koblenz and Landau, Landau, Germany; ^5^Institute for Research on Instruction and Subject-Specific Didactics, PH Schwyz, Goldau, Switzerland

**Keywords:** scientific reasoning, comparison, induction, preschool, labeling

## Abstract

Analogical reasoning by comparison is considered a special case of inductive reasoning, which is fundamental to the scientific method. By reasoning analogically, learners can abstract the underlying commonalities of several entities, thereby ignoring single objects’ superficial features. We tested whether different task environments designed to trigger analogical reasoning by comparison would support preschoolers’ induction of the concept of material kind to predict and explain objects’ floating or sinking as a central aspect of scientific reasoning. Specifically, in two experiments, we investigated whether the number of presented objects (one versus two standards), consisting of a specific material and the labeling of objects with the respective material name, would benefit preschoolers’ material-based inferences. For each item set used in both experiments, we asked the children (*N* = 59 in Experiment 1, *N* = 99 in Experiment 2) to predict an object’s floating or sinking by matching it to the standards and to verbally explain their selections. As expected, we found a significant effect for the number of standards in both experiments on the prediction task, suggesting that children successfully induced the relevance of material kind by comparison. However, labels did not increase the effect of the standards. In Experiment 2, we found that the children could transfer their conceptual knowledge on material kind but that transfer performance did not differ among the task environments. Our findings suggest that tasks inviting analogical reasoning by comparison with two standards are useful for promoting young children’s scientific reasoning.

## Introduction

Analogical reasoning by comparison is assumed to be a crucial mechanism, enabling induction and conceptual learning across different age groups and in a wide range of tasks (Loewenstein and Gentner, 2001; Alfieri et al., 2013; Schalk et al., 2016). According to the theory of structural alignment (Gentner, 2010), analogical reasoning involves individuals’ identification, mapping, and evaluation of the similarities and differences of several entities. This process has benefits for building conceptual knowledge because it supports individuals’ encoding of information, induction and abstraction of categories, and generalization (transfer) of knowledge (Gentner and Smith, 2012; Gentner and Hoyos, 2017). While analogical reasoning by comparison has been investigated in experimental settings on a range of conceptual learning tasks (Alfieri et al., 2013), its contribution to scientific reasoning is rarely considered. We propose that task environments that trigger comparison are relevant to scientific reasoning. Specifically, such environments may facilitate children’s induction of scientific concepts as a basis for predictions and explanations by their encoding of relevant object features.

In the present research, we investigated different task environments that may facilitate preschoolers’ encoding of relevant object features to generate predictions and explanations in the science context of “floating and sinking.” Research with preschoolers across various science contexts has revealed that preschoolers typically hold a variety of naïve conceptions based on irrelevant and perceptually salient features and that these conceptions may affect hypothesis generation (Carey, 2000). For example, when preschoolers are asked to predict the floating or sinking of solid objects and to explain their predictions, they provide explanations such as “light things will float,” “things with holes will sink,” “large things will sink,” or “things with air in them will float” (Penner and Klahr, 1996; Leuchter et al., 2014). Thus, preschoolers’ predictions about whether objects float or sink are typically based on salient features, such as weight, size, and form, rather than on generic and more abstract aspects such as material kind or density. Children’s naïve conceptions prevail despite the fact that they are typically able to name the material of solid objects, such as wood, plastic, or metal (Smith et al., 1985; Dickinson, 1987; Leuchter et al., 2014). Typically, a process of conceptual restructuring is required for children to overcome their naïve conceptions and transform them into scientifically advanced conceptions (Schneider et al., 2012; Leuchter et al., 2014). In two experiments, we investigated whether different task environments intended to trigger analogical reasoning by comparison (i.e., presenting two objects at the same time and labeling them) would improve preschoolers’ predictions and explanations of floating or sinking based on the concept of material kind.

### Scientific Reasoning in Preschoolers

The goals of science education encompass mastery of scientific concepts as well as learning how to engage in scientific reasoning (Driver et al., 2009; Kuhn, 2010; Dunbar and Klahr, 2012; Sandoval et al., 2014). In general terms, scientific reasoning involves individuals’ knowledge-seeking by the application of scientific methods (Klahr and Dunbar, 1988; Klahr, 2000; Piekny and Maehler, 2013). Models of scientific reasoning typically refer to processes of inductive reasoning to explain individuals’ knowledge construction, as well as their hypothesis and inference generation (Zimmerman, 2007; Morris et al., 2012). Inductive reasoning is regarded as a cognitive process that captures how individuals encode information, mentally represent this information, organize information into patterns, and derive inferences (cf. Chinn and Brewer, 2001; Chinn and Malhotra, 2002; Dunbar and Klahr, 2012). With respect to hypothesis generation, Klahr and Dunbar (1988) differentiate between “evoking” and “inducing” a hypothesis. When evoking a hypothesis, individuals retrieve and rely on prior knowledge. When inducing a hypothesis, individuals need to observe and encode data, and to identify patterns, before venturing an initial hypothesis. Therefore, the generation of a hypothesis requires children to encode relevant observations, to identify underlying patterns, and to draw inferences, which may rely in part on prior conceptual knowledge.

Several reviews indicate that even preschoolers can exhibit basic aspects of scientific reasoning (Chinn and Malhotra, 2002; Zimmerman, 2007; van der Graaf et al., 2016). There is substantial evidence indicating that preschoolers can appropriately generate hypotheses, identify common patterns in data, and evaluate presented evidence in specific task contexts. With regard to hypothesis generation, Piekny and Maehler (2013) found that 4- to 6-year-olds were able to construct a hypothesis based on patterns of evidence. If task contexts are kept simple, 5- and 6-year-olds are even able to identify patterns that are more complex and to form various hypotheses (e.g., Sodian et al., 1991; Ruffman et al., 1993). Moreover, an intervention study by Schulz et al. (2007) revealed that 4- to 6-year-olds were not only able to infer the causal structure of events by using experimentally collected patterns of evidence but also to predict the outcome of a novel experiment. Piekny et al. (2014) found that 4- to 6-year-olds were able to evaluate conclusive and partially conclusive evidence correctly, and Koerber et al. (2005) found that the correspondence between preschoolers’ conceptions and presented evidence facilitated evaluation, whereas conflicting conceptions impeded evaluation. Even the 4-year-olds in the study were able to understand that data with perfect covariation could corroborate or disconfirm a causal hypothesis (see also Tullos and Woolley, 2009). Finally, a study by Köksal-Tuncer and Sodian (2018) revealed that 3- to 6-year-olds were not only able to generate hypotheses but also to apply hypothesis-testing strategies when presented with counterevidence.

In this prior research, selection tasks and production tasks were employed to assess young children’s generation of hypotheses in scientific reasoning contexts (Koerber et al., 2005; Piekny and Maehler, 2013; Gropen et al., 2017). Selection tasks provide children with different answer options to a given problem and may be employed to assess children’s spontaneous reasoning with regard to a given scientific reasoning context. By contrast, production tasks require children to come up with solutions themselves on the basis of explicit reasoning. Production tasks have therefore been employed to assess children’s deliberate reasoning based on the production, explanation, and evaluation of arguments (Mercier and Sperber, 2009; Mercier, 2011). For our two experiments, we used a selection task in which we asked children to predict which object would float or sink like one or two other objects (prediction task). We also employed a production task in which we asked children to explain their respective prediction in order to assess their deliberate reasoning (explanation task).

Overall, preschoolers’ success in generating hypotheses is typically assessed in terms of the adequacy of their predictions and explanations based on given data patterns. However, research has rarely focused on the specific task conditions supporting the formation of predictions and explanations. We suggest that research on analogical reasoning by comparison (Gentner and Smith, 2012; Alfieri et al., 2013) may provide insights and that task contexts triggering analogical reasoning may benefit the performance of young children in a scientific reasoning context.

### Analogical Reasoning by Comparison

Analogical reasoning can be understood as a special case of inductive reasoning (Holland et al., 1989; Holyoak, 2005) because it refers to individuals’ ability to integrally encode commonalities and differences across a variety of entities and situations, represent and re-represent this information, and draw inferences (Gentner, 2016). Analogical reasoning has been demonstrated to be fundamental to cognitive development and conceptual learning, such as spatial orientation, word learning, learning of principles, and social comparison (for summaries, see Holyoak, 2005; Gentner, 2010; Gentner and Hoyos, 2017). One strategy for promoting analogical reasoning is to invite comparisons. According to the theory of structural alignment, comparison involves the retrieval of relevant information from the long-term memory, the mapping of commonalities and differences of two (or more) presented entities or cases (thereby inducing an abstracted schema), and the projection of inferences based on this mapping (Gentner and Smith, 2012). The abstracted schema will be more general than the analogs because inferences are formed on the basis of aligned similarities and differences, leading to a merged representation—a process of “relational pattern completion” (Gentner and Smith, 2012).

Schema abstraction can be supported by simultaneously presenting two entities or examples (Gentner and Medina, 1998; Gentner and Namy, 1999; Gentner et al., 2007; Kurtz and Loewenstein, 2007; Gentner, 2010; Alfieri et al., 2013; Christie, 2020). Specifically, Gentner and Namy (1999) argue that the presentation of two standards may “promote the discovery of relatively abstract relational commonalities that could characterize the category being learned” (p. 506). Christie (2020) provides empirical support to this claim. She reports on a series of studies in which young children successfully learned categories when presented with multiple exemplars. In addition, Christie and Gentner (2010) showed that 4-year-olds were able to recognize similarity in relations (above, under) in pictures of animals in different positions by making correct choices in a selection task. Gentner and Rattermann (1991) showed, in a more complex task, that 3-year-olds were unable to carry out relational matches without additional support, evidenced by choosing an object match instead of a relational match, but that 5-year-olds were successful in this task. Despite positive evidence from prompting comparisons even in young children, presenting two cases, examples, entities, or situations simultaneously may not always be sufficient for learners to recognize similarities (Kurtz et al., 2001). In particular, it has been shown that young children benefit from additional prompts, such as the use of common labels, when presented with two objects to be compared (Gentner, 2010; Christie, 2020).

### Using Language to Promote Comparison

Labels and other types of verbal scaffold often facilitate analogical reasoning by comparison (Alfieri et al., 2013). Gentner and Namy (1999, 2006), Namy and Gentner (2002), Gentner (2010, 2016), and Hespos et al. (2020) argue that language, in general, plays a decisive role in triggering analogical reasoning by comparison even in young children. Language and structure-mapping are suggested to bootstrap each other, mutually influencing cognitive and conceptual development in young and older children as well as adults (Gentner, 2010; Christie, 2020). As Gentner and Rattermann (1991, p. 260) put it, “a word can function as a promissory note, signaling subtle commonalities that the child does not yet perceive.” Gentner (2010) proposes four ways in which language and structure-mapping interact: (1) Common labels invite comparison and abstraction by highlighting similarity across entities; (2) the naming of entities promotes reification since it preserves abstraction linguistically; (3) the naming of entities promotes uniform relational encoding; and (4) the use of linguistic structures invites the construction of conceptual structures. In the present research, we focus on the function of labels to invite and trigger comparison.

In a series of experiments, Gentner et al. (2007) investigated the use of common labels for triggering analogical reasoning by comparison. For example, Namy and Gentner (2002) tested two groups of 4-year-olds using a forced-choice match-to-sample task in which the children had to extend the label of one object (the so-called *standard*) to one of two other objects. In the no-comparison group, the experimenter labeled a single standard (e.g., a picture of an apple) with a made-up name (e.g., blicket). The children had to decide which one of the two other objects, the taxonomic item (banana) or the perceptually similar item (balloon), would have the same name as the standard. In the comparison group, the task was the same except that the experimenter showed and labeled pictures of two taxonomically related standards (e.g., an apple and a pear) with the same made-up name. Namy and Gentner found that preschoolers’ correct taxonomic choices for this task increased when two standards were presented rather than just one standard (see also Gentner and Namy, 1999). Importantly, children were even more likely to make taxonomic choices when the two standards were labeled with the same noun. By contrast, when the two standards received different labels, children did not engage in comparison, as indicated by their increased selection of the perceptually similar but taxonomically unrelated item.

This effect of labeling not only holds for children’s category learning but also for their generalization of properties across entities. That is, the presence of a common label enhances young children’s willingness to make inductive inferences between entities (e.g., Gelman and Markman, 1986; Gelman et al., 1986; Davidson and Gelman, 1990; Saalbach et al., 2012). For example, Gelman and Markman (1986) found that preschoolers can generalize properties across members of the same category when category membership is labeled with the same noun, but not when it is unlabeled. Thus, linguistic labels can serve as simple scaffolds to trigger young children’s comparison processes since they suggest similarity between two items.

Importantly, these beneficial effects of labels have typically been found in tasks that require only a low degree of prior knowledge in young children (Christie, 2020). In our study, we investigated the role of labels in a domain in which learners start with some degree of prior conceptual knowledge. Specifically, we propose that labels of material kind (e.g., “this is made of wood”) will function as a cue at a superordinate level. Material labels provide rich associations with unique properties of objects, such as their texture and specific weight, as well as their empirically observable behavior of floating or sinking in water. From early on, children learn that common labels are used for things that are alike. If labels are used in an instructional context, they can elicit comparison of the respective objects and thereby highlight the relevant (underlying) commonality of different yet related entities even in the absence of perceptual similarity (Gentner, 2010). In several studies it has been shown that nouns provide information about classes of objects better than verbs or adjectives do (Arunachalam and Waxman, 2010; Graham et al., 2012). In addition, Johanson and Papafragou (2016) found that labeling using nouns works with similar success as labeling using facts (e.g., descriptions of properties) in ambiguous situations. In our two experiments, we systematically varied the use of superordinate-level nouns when labeling objects to promote comparison.

### The Present Research

We tested whether different task environments designed to trigger analogical reasoning by comparison with or without labeling the respective objects would support preschoolers’ induction of the concept of material kind to predict and explain objects’ floating or sinking. Specifically, 4- to 7-year-old preschoolers were exposed to sets of material within a forced-choice match-to-sample task with variations in the number of standard objects and the use of superordinate labels. On the basis of Gentner (2010) and the results from Namy and Gentner (2002), we expected that triggering comparison via the use of linguistic labels would amplify the effect of presenting two objects simultaneously. In order to probe the effect of labeling on performance, we varied the extent to which the labels were employed across our two experiments.

## Experiment 1

In Experiment 1, we crossed two factors: the number of standard objects and the use of material labels. Specifically, we presented either one standard or two standards to test the effect of comparison on children’s predictions and explanations, and we either labeled the presented objects or did not label them at all in order to test the additional effect of labeling on the potential benefits of comparison. The labels referred to material kind and therefore served as superordinate category labels indicating common properties of the presented objects. Consequently, it was possible that they might amplify children’s perception of the similarities between the presented objects in conditions with two standards. Before the children were randomly assigned to the four conditions of Experiment 1, their prior knowledge was assessed with a pretest and a baseline assessment.

Our research questions and hypotheses were as follows:

(1) Will preschoolers induce the concept of material kind when analogical reasoning by comparison is triggered by presenting two standards with the same floating behavior?
*Hypothesis (1)*: Preschoolers in conditions with two standards will outperform preschoolers in conditions with one standard on the Prediction and Explanation Tasks.
(2) Does the use of material labels facilitate preschoolers’ induction of the concept of material kind?
*Hypothesis (2)*: The use of material labels will improve performance in the Prediction and Explanation Tasks in the condition with two standards, as indicated by an interaction effect of Labeling and Number of Standards.

### Method

#### Participants

Fifty-nine preschoolers from a major German city who had German as a first language and a mean age of 5 years, 3 months (min. = 4 years, 11 months; max. = 6 years) participated in this study (testing was in German). They were recruited through preschools. Parental consent on participation was collected for all children. The children came from middle-class families living in urban and suburban areas.

#### Design

In a 2 × 2 between-groups design, we tested the importance of triggering a comparison (one standard or two standards) and the benefit of labeling standards with the respective material label (unlabeled versus labeled). Specifically, the four conditions were (1) One Unlabeled Standard (one_unlabeled); (2) One Labeled Standard (one_labeled); (3) Two Unlabeled Standards (two_unlabeled); (4) Two Labeled Standards (two_labeled). In the unlabeled conditions, the standards were referred to as *“this”/“these”*; in the labeled conditions, the standards’ material was named (e.g., *“this/these is/are made of wood”*). In all conditions, children had to predict (Prediction Task) and explain (Explanation Task) which of the four selection objects would float or sink like the standards. Table 1 gives an overview of the experimental conditions and the respective instructions.

**TABLE 1:** Conditions and instructions in Experiment 1.

	**One Standard**	**Two Standards**
Unlabeled	Look, this one floats/sinks in water. Which of these also floats/sinks just like this one?	Look, these two float/sink in water. Which of these also floats/sinks just like these ones?
Labeled (example: wood)	Look, this is made out of wood and it floats in water. Which of these also floats just like this one: this one made of wood, this one made of metal, this one made of metal, or this one made of glass?	Look, this one is made out of wood and it floats in water. And this one is also made out of wood and it floats, too. Which of these also floats just like these ones: this one made of wood, this one made of clay, this one made of clay, or this one made of metal?

Before the children were randomized to these four conditions, all children participated in a pretest and a baseline assessment. In the pretest, children had to match objects made of the same material (Matching Task) and to name the objects’ material (Labeling Task). Subsequently, in the baseline assessment, children were presented with one standard that was not labeled and four selection objects. As in the four conditions described above, children had to predict (Prediction Task) and explain (Explanation Task) which of the four selection objects would float or sink like the standard.

#### Tasks and Procedure

All participants were tested individually in a quiet room in their preschools by an experimenter blind to the hypotheses. Testing began with the pretest and the baseline assessment, comprising the Prediction and Explanation Tasks; afterward, the children took the Prediction and Explanation Tasks with different materials, depending on the conditions.

##### Pretest: Matching/labeling task

Two tasks were employed to assess the children’s prior knowledge of materials and their labels. The children were presented with a total of 16 objects made of eight different materials (wood, stone, metal, plastic, Styrofoam, wax, glass, clay), each with two different shapes (e.g., a wooden block and a wooden spoon). In the Matching Task, the children were asked to match pairs of objects: *“Find the two things that belong together and put them together on the table.”* After a child had matched all the objects, the experimenter rearranged them in pairs by material regardless of how the children had arranged them in the Matching Task. In the Labeling Task, the children were asked to name the material: *“Tell us what the objects are made of*.*”* The experimenter did not use the term “material.” The Matching Task was scored with respect to the successful matching of objects according to their material, with one point assigned for each correct material-based match of two objects (range of scores 0–8). The Labeling Task was scored with one point for each correct material label (range of scores 0–8).

##### Baseline assessment: Prediction and explanation task

The baseline assessment served to measure how children would predict and explain objects’ floating or sinking if they were only provided with a single standard that was not labeled, i.e., a task environment without elements to support comparison. Specifically, we employed six object sets in the baseline assessment. All objects in these sets were different from the objects used in the pretest. In each set, one object of a specific material served as the standard; four objects served as the selection items. In each object set, only one of the selection objects was made of the same material as the standard, but it always had a different shape and size. The other three selection objects were distractors that were selected on the basis of children’s typical misconceptions (Hardy et al., 2006). Of the three distractors, there was one with the same shape as the standard. The other two distractors had a salient size or weight. That is, if the standards floated, we used extremely light and/or small selection objects (e.g., a small needle), whereas if the standards sank, we used extremely heavy and/or large selection objects (e.g., a large piece of wax). Only the selection object made of the same material as the standards sank/floated like the standards. Half of the sets had a standard made of material that floats in water (wood, wax, Styrofoam), and the other half had a standard made of material that sinks in water (metal, plastic, clay). Table 2 provides an overview of all objects sets used in the baseline assessment and the conditions of Experiment 1 and Experiment 2.

**TABLE 2:** Object sets in Experiments 1, 2.

	**Baseline**		**Conditions**	
**Object Set**	**Standard**	**Choices**	**Standard**	**Choices**
Wood	Wooden plate	Wooden spoon Metal plate Metal needle Glass marble	Wooden star (wooden ball)	Wooden cube Clay star Clay marble Metal fragment
Clay	Clay fragment	Clay puppet Wax fragment Wax block Cork block	Clay marble (clay stick)	Clay fragment Styrofoam marble Styrofoam stick Cork block
Plastic	Plastic knife	Plastic plate Wooden knife Wooden block Wax fragment	Plastic ruler (plastic plate)	Plastic spoon Wooden ruler Wooden plate Styrofoam ring
Wax	Wax sphere	Wax block Glass sphere Glass marble Metal nut	Wax star (wax fragment)	Wax candle Clay star Clay fragment Glass marble
Metal	Metal ring	Metal marble Wooden ring Wooden button Wax block	Metal spoon (metal ball)	Metal nut Wooden spoon Wooden block Wax candle
Styrofoam	Styrofoam pyramid	Styrofoam block Metal needle Metal nut Clay pyramid	Styrofoam sphere (Styrofoam cube)	Styrofoam plate Glass cube Glass sphere Metal needle

In the Prediction Task the experimenter first took the standard and said “*Look, this floats/sinks in water.*” The experimenter then immersed the standard in a water basin, and the child observed whether the object sank or rose to the top. The experimenter then positioned this object above the four selection objects and asked “*Which of these also floats/sinks, just like this one here? This one, this one, this one, or this one?*” while pointing at the four selection items. The child then selected one of four objects that would float/sink just like the standard. The children received one point for choosing the selection object of the same material as the presented standard and zero points for choosing one of the other three selection objects (i.e., the range of possible scores for the Prediction Task was 0–6).

The Explanation Task followed immediately after the child had made their prediction. That is, once the child had chosen one of the four selection objects, the experimenter asked the child to explain their prediction by asking, *“Why do you think so?”* For every set, we coded whether the answers referred to material kind. If the child referred to the material or an according quality at least once (e.g., *“because it is made out of wood,” “because it is made out of the same stuff,” “because it is just the same”*), the child received one point (i.e., the range of possible scores for the Explanation Task was 0–6).

##### Conditions: Prediction and explanation task

**FIGURE 1:**
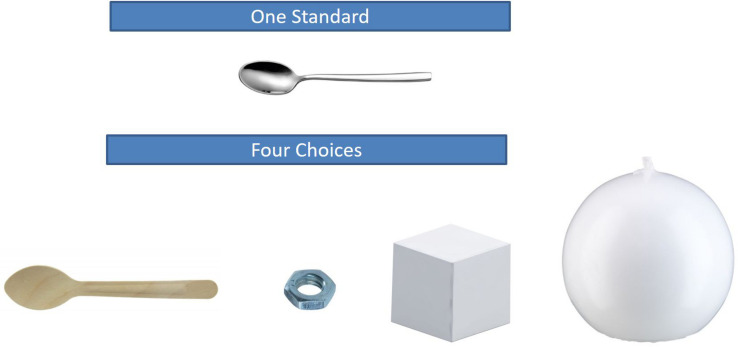
Object set for prediction task in Experiment 1 and Experiment 2 (one standard).

**FIGURE 2:**
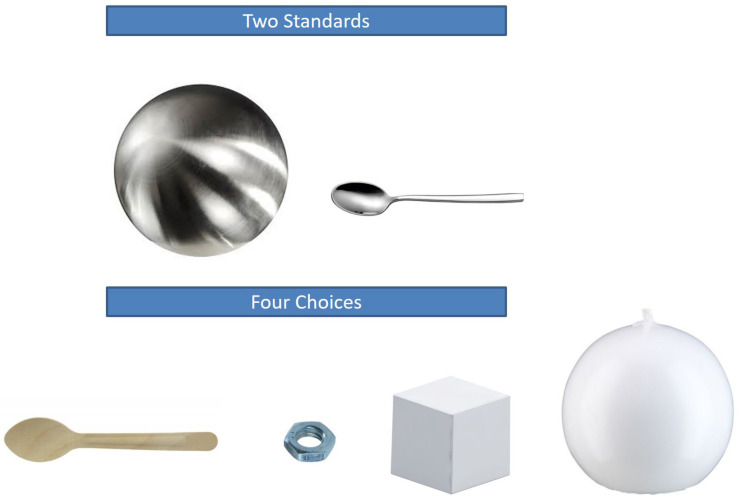
Object set for prediction task in Experiment 1 and Experiment 2 (two standards).

After the baseline assessment, the four different conditions followed. The materials used in the conditions were composed in a way that was similar to the baseline assessment. That is, we developed six novel object sets following the same logic as described above. However, all objects had different shapes than the objects used in the baseline assessment (and than the objects used in the pretest), and we chose a second standard for each set to be presented in the conditions with two standards (see Figures 1, 2 for object sets in the one-standard condition and the two-standard condition, respectively).

The instructions and the number of standards varied for the four conditions (see Table 1). The children saw either one or two standards, and the objects of a set were either labeled or unlabeled. In the labeled conditions, the standards were labeled according to their material after their floating behavior had been shown (e.g., *“Look, this one is made out of wood and it floats in water. And this one is also made out of wood and it floats, too*” in the two-standard condition). To increase the salience of labels, the labeling was not only applied to the standards but also to all selection objects.

In the Prediction Task, the children were asked to choose one of the selection objects that would show the same floating behavior (*“Which of these also floats in water, just like these two? This one made out of iron, this one made out of wood, this one made out of glass, or this one made out of clay?”*). Afterward, the children were asked to explain their selection (Explanation Task). The Prediction and the Explanation Tasks were scored as described for the baseline assessment (i.e., the range of scores was 0–6). In all conditions, the children were allowed to touch the objects and hold them in their hands, but they were not allowed to put them into the water. Only the experimenter immersed the standards into water for the children to observe. If the children expressed a wish to immerse objects into the water, they were told that they could do so after the experiment was finished. In all phases of the experiment, the children were praised for their active participation; however, no feedback was given concerning the accuracy of their replies.

### Results

Table 3 presents the means and standard deviations of the Matching Task and the Labeling Task (pretest), as well as of the Prediction Task and the Explanation Task for the baseline assessment and four conditions. The scores of the Prediction and the Explanation Tasks of the baseline assessment were employed as covariates in the respective task analyses of children’s performance in the different conditions.

**TABLE 3:** Means (standard deviations) of pretest, prediction task, and explanation task by condition in Experiment 1.

	**Pretest**		**Prediction Task**		**Explanation Task**		
**Condition**	**Matching**	**Labeling**	**Baseline**	**Experimental**	**Baseline**	**Experimental**	***N***
One_unlabeled	5.60 (1.55)	3.07 (2.05)	2.13 (1.35)	1.73 (1.43)	2.0 (1.56)	1.13 (1.68)	15
One_labeled	6.07 (1.07)	2.78 (1.31)	1.43 (1.22)	3.21 (1.58)	0.72 (0.92)	1.86 (2.14)	14
Two_unlabeled	6.07 (1.39)	2.93 (1.83)	1.73 (1.39)	2.67 (1.59)	0.54 (0.52)	0.80 (1.20)	15
Two_labeled	6.20 (1.57)	3.00 (1.60)	1.93 (0.96)	4.13 (1.81)	1.07 (0.96)	2.80 (2.31)	15

Before presenting the results with regard to our hypotheses, we present the results of the preliminary analyses testing whether there were significant differences across conditions on the two pretest tasks and the children’s performance in the Prediction Task and Explanation Task in the baseline assessment. One-way ANOVAs showed no significant differences across the experimental conditions for the pretests (Matching Task: *p* = 0.67, η_p_^2^ = 0.028; Labeling Task: *p* = 0.98, η_p_^2^ = 0.004). The Labeling Task (36% correct) was more difficult than the Matching Task (74% correct). There was also no significant difference in the Prediction Task for the baseline assessment across conditions (*p* = 0.47, η_p_^2^ = 0.04). There was, however, a significant difference in the Explanation Task of the baseline assessment across conditions, *F*(3,55) = 5.68, *p* = 0.002, η_p_^2^ = 0.24. Follow-up analyses of the Explanation Task indicated that children in the one_unlabeled condition had significantly higher scores than children in the one_labeled and the two_unlabeled conditions (see Table 3).

To test Hypotheses 1 and 2 (i.e., better performance in the two-standard conditions and an interaction effect of Number of Standards and Labeling), a 2 × 2 ANCOVA with Number of Standards (one vs. two) and Labeling (unlabeled vs. labeled) and the Prediction Task performance as the dependent measure was computed, using the performance in the Prediction Task in the baseline assessment as covariate. The covariate significantly predicted the dependent measure, *F*(1,54) = 4.35, *p* = 0.04, η_p_^2^ = 07. As expected, we found a significant effect of the Number of Standards, *F*(1,54) = 4.97, *p* = 0.03, with overall higher mean accuracy in the two-standard condition, *M* = 3.40 (*SD* = 1.83) than in the one-standard condition, *M* = 2.45 (*SD* = 1.66). However, the size of the effect was rather small (η_p_^2^ = 0.08). We also found a significant but small main effect of Labeling, *F*(1,54) = 4.21, *p* = 0.01, η_p_^2^ = 0.04, with higher mean accuracy in the labeled conditions, *M* = 3.69 (*SD* = 1.73) than in the unlabeled conditions, *M* = 2.20 (*SD* = 1.56). Contrary our expectation, there was no interaction of Number of Standards × Labeling, *p* = 0.69, η_p_^2^ = 0.003.

We performed the same analyses with the performance in the Explanation Task as a dependent measure, using performance in the Explanations Task in the baseline assessment as covariate. The covariate significantly predicted this dependent measure, *F*(1,54) = 6.26, *p* = 0.015, η_p_^2^ = 0.10. We found only a significant effect of Labeling, *F*(1, 54) = 11.00, *p* = 0.002, η_p_^2^ = 0.17, with *M* = 2.34 (*SD* = 2.24) for the labeled conditions and *M* = 0.97 (*SD* = 1.45) for the unlabeled conditions. Contrary to our hypotheses, however, there was no significant main effect of Number of Standards, *p* = 0.20, η_p_^2^ = 0.03, and no significant Number of Standards × Labeling interaction, *p* = 0.82, η_p_^2^ = 0.001.

In exploratory *post hoc* analyses, we checked whether age contributed to the reported effects. We did not find significant differences between younger children (≤64 months, *N* = 36) and older children (>65 months, *N* = 37) in the Prediction Task, *M* = 3.0 (*SD* = 1.85), *M* = 3.13 (*SD* = 1.62), respectively, *p* = 0.79, η_p_^2^ = 0.01, or in the Explanation Task, *M* = 1.39 (*SD* = 1.92), *M* = 1.27 (*SD* = 1.90), respectively, *p* = 0.67, η_p_^2^ = 0.01.

### Discussion of Experiment 1

In Experiment 1, we investigated whether the presentation of two standards of the same material would support children in relying on the concept of material kind when predicting and explaining objects’ floating or sinking, and whether the labeling of objects with their respective materials would increase children’s ability to induce the concept of material kind. We found that preschoolers were indeed more likely to base their predictions on the concept of material kind when comparison was triggered by the presentation of two standard objects, with an overall small but significant effect. When presented with two standard objects, children decreased their reliance on irrelevant features, such as shape or size, when predicting floating or sinking. However, our hypothesis that the effect of comparison would be intensified by presenting labels was not confirmed. While we found a main effect of Labeling in the Prediction Task, we did not find a significant interaction between the factors Number of Standards and Labeling. Unexpectedly, even in the one-standard condition, the children were more likely to choose objects with the same material if they were provided with objects’ material labels. This finding suggests that children in the one-standard condition used the common label of the standard and the same-material item of the selection objects to derive conclusions with respect to their underlying commonalities.

In the Explanation Task, we found an effect only for Labeling. As for the Prediction Task, the children showed a significantly higher tendency to refer to material when explaining their choices in the conditions when labels were used. In contrast to the results of the Prediction Task and in contrast to our hypothesis, however, there was no effect of the Number of Standards factor. It would appear that the children were able to base their predictions on material kind in conditions with two standards, but they did not explicate their intuitive knowledge when prompted for explanations unless they were supported by labels. On average, the solution rates for explanations were lower than for predictions. Therefore, one might speculate that this task placed a higher demand on children with regard to the retrieval of conceptual knowledge. A significant group difference had already been detected in the Explanation Task of the baseline assessment prior to the experimental variation, and this *a priori* difference may have diluted the effect of producing explanations in the experimental conditions. We accounted for this difference by using the baseline performance in the Explanation Task as a covariate. Nevertheless, this unexpected baseline difference may have biased performance in the Explanation Task. Thus, these results should be treated with caution.

How may we explain the effect of labeling on children’s performance? First, it is likely that the labels presented in Experiment 1 elicited comparison processes that directed the children’s attention to the objects’ material, especially because material names were used to label all objects. While developmental research has shown that basic-category labels prompt analogical reasoning by comparison in children from an early age (Gelman and Markman, 1986; Davidson and Gelman, 1990; Nguyen and Gelman, 2012; Childers, 2020), Experiment 1 showed that material labels that provide superordinate information may also support inductive reasoning to derive predictions and explanations for floating and sinking. Second, using material labels may activate specific prior conceptual knowledge of material kind. For example, children may know that “wood” refers to the specific quality that some floating objects are made of. Thus, the children may have been more likely to pick wooden objects due to their prior experience. As we applied material labels to the standards and to all selection objects, the children’s attention was drawn to material kind as a quality of all the presented objects. Thus, the activation of prior knowledge may have been especially strong. Finally, the effects of Number of Standards and Labeling in the Prediction Task were rather small. In contrast to Namy and Gentner’s (2002) study, we used a baseline assessment of children’s initial performance. Using this baseline performance as a covariate increases the power to detect effects. Moreover, Namy and Gentner investigated basic-category learning, whereas our study employed tasks in a scientific reasoning context. It is likely that this type of task led to rather small experimental effects since conceptual restructuring in science is difficult to achieve with short-term instructional interventions (Schneider et al., 2012). Specifically, the induction of a concept of material kind may be regarded as a process of initial conceptual restructuring. Since we chose our selection objects in the Prediction and Explanation Tasks on the basis of well-known misconceptions about floating and sinking at preschool and elementary school age (Leuchter et al., 2014), this task requires children to inhibit answers due to misconceptions to make the correct prediction and provide the correct explanation.

To evaluate whether an effect of labeling is indeed due to the activation of prior conceptual knowledge, we contrasted the use of real material labels and made-up labels in Experiment 2. If the labeling effect were due to the activation of prior conceptual knowledge, then we would find effects only for real labels and not for made-up labels To diminish the effects of drawing attention to material kind as a dimension of all objects in a set, we labeled only the standards and not the selection objects.

## Experiment 2

As in Experiment 1, we varied whether one or two standards were presented and whether the standards were unlabeled (i.e., *“this/these”*) or labeled with respect to their material (e.g., *“this/these one/s is/are made of wood”*). In addition to the use of real material labels, we included two conditions in which we used made-up labels (e.g., *“these ones are made of idoform”*). If the labeling effect in Experiment 1 was due to the triggering of analogical reasoning by comparison, then the effect would appear only in the two-standard conditions. If it was due to the triggering of prior conceptual knowledge by the use of the real material labels, then the effect would only appear in the labeled conditions with real material names. In addition to the Prediction and the Explanation Tasks of Experiment 1, we assessed whether there was evidence of conceptual knowledge transfer with regard to predicting whether novel objects would float or sink. We therefore employed a test of conceptual knowledge immediately before the baseline assessment (transfer pretest) and after the children had completed the conditions (transfer posttest).

Our research questions and hypotheses were as follows:

(3) Will preschoolers induce the concept of material kind when analogical reasoning by comparison is triggered by the presentation of two standards with the same floating behavior?
*Hypothesis (3)*: In conditions with two standards, preschoolers will outperform participants in conditions with one standard on the Prediction and the Explanation Tasks.
(4) Does the use of material labels trigger analogical reasoning by comparison?
*Hypothesis (4)*: The use of real material labels will improve performance in the two-standard condition in the Prediction and Explanation Tasks, but not the use of made-up labels.
(5) Will the intervention lead to conceptual knowledge transfer?
*Hypothesis (5)*: There will be knowledge transfer in the two-standard conditions.

### Method

#### Participants

Ninety-nine children from Central Switzerland with German as a first language participated (testing was in German). They were recruited with parental consent through preschools. The mean age was 5 years, 8 months, ranging from 4 years, 4 months to 7 years. The children were mostly from middle-class families living in suburban areas.

#### Design

Experiment 2 was based on a 2 × 3 between-groups design, including a baseline assessment as in Experiment 1. After the pretest and the baseline assessment, the preschoolers took a conceptual knowledge transfer test (transfer pretest). They then participated in one of six conditions: (1) One Unlabeled Standard (one_unlabeled), (2) One Standard Labeled with Real Material Label (one_real), (3) One Standard Labeled with Made-up Material Label (one_ made-up), (4) Two Unlabeled Standards (two_unlabeled), (5) Two Standards Labeled with Real Material Label (two_real), or (6) Two Standards Labeled with Made-up Material Label (two_ made-up). After the conditions, the conceptual knowledge transfer test was again presented (transfer posttest). Table 4 gives an overview of the different conditions and the respective instructions.

**TABLE 4:** Conditions and instructions in Experiment 2.

	**One Standard**	**Two Standards**
Unlabeled	Look, this one floats/sinks in water. Which of these also floats/sinks just like this one?	Look, these two float/sink in water. Which of these also floats/sinks just like these two?
Real Label (example: wood)	Look, this one is made out of wood and it floats in water. Which of these also floats just like this one?	Look, this one is made out of wood and it floats in water. And this other one is also made out of wood and it floats, too. Which of these also floats just like these two?
Made-up Label	Look, this one is made out of feb and it floats in water. Which of these also floats just like this one?	Look, this one is made out of feb and it floats in water. And this other one is also made out of feb and it floats, too. Which of these also floats just like these two?

#### Tasks and Procedure

The material, tasks, and procedure employed in Experiment 2 were largely similar to those of Experiment 1. In addition, we employed a conceptual knowledge transfer test that was first conducted immediately before the baseline assessment and then repeated after the children had solved the tasks in the different conditions.

##### Pretest: Matching/labeling task

The material and procedure for these tasks were the same as in Experiment 1.

##### Baseline assessment and conditions: prediction and explanation task

The object sets (see Table 2) and procedure were the same as in Experiment 1 for the baseline assessment and for the unlabeled conditions (one_unlabeled, two_unlabeled). For the labeled conditions, the procedure differed from Experiment 1 in the following way. In the conditions using real material labels (one_real, two_real), the standards were labeled while their floating behavior was demonstrated (e.g., *“Look, this is made out of wood and it floats in water. And this is also made out of wood and it floats, too.”*). Afterward, the children were asked to select one of the selection objects (*“Which of these also floats/sinks in water, just like those two? This one, this one, this one, or this one?”*). Thus, in contrast to the procedure in Experiment 1, we applied the labels only to the standards and not to the four selection objects. In the conditions with made-up labels (one_made-up, two_made-up), the procedure was the same as in the real material label conditions except that the real material labels were replaced with made-up labels. For all objects sets, the children had to choose one of the selection objects (Prediction Task) and to explain their selection (Explanation Task). These two tasks were scored as in Experiment 1.

##### Test on conceptual knowledge transfer of floating and sinking

We designed a conceptual knowledge test to measure potential knowledge transfer. This test also included Prediction and Explanation Tasks. In the Transfer Prediction Task, the children were asked to predict whether a presented object would float or sink in water. In the Transfer Explanation Task, the children were asked to verbally explain their answers. Five objects were used, all consisting of material that was also used in the object sets for the baseline assessment and in the different conditions. However, the objects in the transfer tasks had novel shapes and were selected because they represented common misconceptions based on the perceptual qualities of size, weight, or shape: A large and heavy wooden block, a thin wooden board with holes, a metal cube, a small metal needle, and a large block of Styrofoam (see also Leuchter et al., 2014, for a similar task). When predicting whether such objects float or sink in water, children typically refer to their size, weight, or shape in their explanations rather than to their material. Each object was first shown to the child, and the child was asked to touch it and to hold it. A container with water was placed onto the table next to the objects but the children were only allowed to put the objects into it after all tasks were finished. In the Transfer Prediction Task, the child was first asked *“Does this float or sink in water?”* In the immediately following Transfer Explanation Task, the child was asked *“Why do you think so?”* In the Transfer Prediction Task, the children received one point for a correct prediction (i.e., range of scores 0 – 5). In the Transfer Explanation Task, the children received one point if they provided a correct explanation with regard to material kind (i.e., range of scores 0–5).

### Results

Table 5 presents the means and standard deviations of the six conditions for the pretest scores (Matching Task, Labeling Task), the Prediction Task, and the Explanation Task in the baseline assessment and the different conditions. Table 6 presents the respective descriptive values for the pre- and posttest scores of the Transfer Prediction Task and the Transfer Explanation Task.

**TABLE 5:** Means (standard deviations) of pretest, prediction task, and explanation task by condition in Experiment 2.

	**Pretest**		**Prediction Task**		**Explanation Task**		
**Condition**	**Matching**	**Labeling**	**Baseline**	**Experimental**	**Baseline**	**Experimental**	***N***
One_unlabeled	5.65 (1.79)	1.78 (1.21)	2.56 (1.54)	2.67 (2.12)	1.55 (1.95)	1.55 (1.85)	18
One_real	4.56 (2.00)	2.17 (2.12)	2.72 (1.81)	3.39 (2.15)	1.72 (2.27)	2.11 (2.35)	18
One_made-up	5.73 (2.22)	2.73 (1.62)	1.87 (2.03)	2.13 (2.23)	1.53 (2.09)	2.27 (2.49)	15
Two_unlabeled	6.11 (1.90)	2.67 (1.85)	2.89 (1.60)	3.94 (2.41)	2.22 (2.12)	3.33 (2.50)	18
Two_real	6.18 (1.13)	2.41 (1.18)	2.24 (1.79)	3.65 (2.09)	1.41 (1.94)	2.53 (2.53)	17
Two_made-up	5.46 (2.50)	2.54 (1.90)	2.54 (2.07)	3.77 (2.13)	1.69 (1.93)	2.85 (2.48)	13

**TABLE 6:** Means (standard deviations) of conceptual knowledge transfer by condition in Experiment 2.

	**Transfer Prediction Task**		**Transfer Explanation Task**		
**Condition**	**Pretest**	**Posttest**	**Pretest**	**Posttest**	***N***
One_unlabeled	3.11 (0.96)	3.39 (1.29)	0.67 (1.33)	1.05 (1.43)	18
One_real	3.61 (0.85)	3.72 (0.75)	0.94 (1.39)	1.11 (1.45)	18
One_made-up	3.13 (0.91)	3.07 (1.10)	0.53 (1.06)	0.53 (0.91)	15
Two_unlabeled	3.39 (1.04)	3.61 (0.85)	0.67 (1.19)	1.44 (1.69)	18
Two_real	3.00 (1.06)	3.05 (0.97)	0.41 (1.00)	0.65 (0.86)	17
Two_made-up	3.54 (0.97)	3.46 (1.27)	1.08 (1.04)	1.54 (2.02)	13

In preliminary analyses, we checked whether the children differed among conditions with respect to their pretest and baseline assessment performances using one-way ANOVAs. There were no significant group differences (Matching Task: *p* = 0.16, η_p_^2^ = 0.08; Labeling Task: *p* = 0.56, η_p_^2^ = 0.04; baseline Prediction Task: *p* = 0.64, η_p_^2^ = 0.035; baseline Explanation Task: *p* = 0.89, η_p_^2^ = 0.018; pretest Transfer Prediction Task: *p* = 0.36, η_p_^2^ = 0.056; pretest Transfer Explanation Task: *p* = 0.64, η_p_^2^ = 0.035).

To test our hypotheses derived from research questions 3 and 4 (i.e., better performance in the two-standard conditions and an improvement in performance in the two-standard condition with real labels), we conducted a 2 × 3 ANCOVA with Number of Standards (one vs. two) and Labeling (no material label vs. real material label vs. made-up label) as between-subject factors, and performance in the Prediction Task in different conditions as the dependent measures, including the baseline Prediction Task performance as covariate. The covariate predicted performance in the Prediction Task significantly, *F*(1,92) = 92.77, *p* = 0.001, η_p_^2^ = 0.50. As expected, we found a main effect for Number of Standards, *F*(1,92) = 8.28, *p* = 0.005, with higher means in the conditions with two standards, *M* = 3.79 (*SD* = 2.18) than in the conditions with one standard, *M* = 2.76 (*SD* = 2.18). However, the effect size was rather small (η_p_^2^ = 0.08). However, we did not find an effect of Labeling, *p* = 0.49, η_p_^2^ = 0.01, nor an interaction of Number of Standards × Labeling, *p* = 0.87, η_p_^2^ = 0.003. Overall, the results suggest that presenting two objects of the same material benefits children’s induction of the concept of material kind. Labeling the material of the standards did not cause significant performance differences, regardless of whether real or made-up labels were used or whether labels were combined with the presentation of two objects.

For the Explanation Task performance, we conducted the same 2 × 3 ANCOVA as for the Prediction Task. We found a similar pattern. There was a significant effect for Number of Standards, *F*(1,92) = 6.16, *p* = 0.015, η_p_^2^ = 0.063, with *M* = 2.92 (*SD* = 2.47) for the two-standard conditions and *M* = 1.96 (*SD* = 2.21) for one-standard conditions. This finding suggests that preschoolers were more likely to explain their choices by reference to material kind when two standards were presented than when only one standard was given. There was no effect for Labeling (*p* = 0.66, η_p_^2^ = 0.01) and no Number of Standards × Labeling interaction (*p* = 0.60, η_p_^2^ = 0.011). The covariate predicted performance in the Explanation Task significantly, *F*(1,92) = 127.67, *p* < 0.001, η_p_^2^ = 0.58. In additional exploratory analyses, we found no significant differences between the age groups of children younger than 60 months (*N* = 11), between 61 and 72 months (*N* = 51), and more than 72 months (*N* = 20) in analyses of covariance with the Prediction Task, *M* = 2.91 (*SD* = 2.43), *M* = 3.12 (*SD* = 2.12), *M* = 3.95 (*SD* = 2.23), respectively, *p* = 0.49, η_p_^2^ = 0.02, or the Explanation Task, *M* = 0.45 (*SD* = 0.82), *M* = 0.94 (*SD* = 1.45), *M* = 1.45 (*SD* = 1.57), respectively, *p* = 0.65, η_p_^2^ = 0.01.

Finally, to test research question 5 (i.e., whether the conditions increased conceptual knowledge transfer), we tested performance in the Transfer Prediction and Explanation Tasks. First, we conducted a 2 × 2 × 3 repeated measurement ANOVA with the pretest and posttest scores for the Transfer Prediction Task as within-subjects factors and Number of Standards (one vs. two) and Labeling (unlabeled vs. real material label vs. made-up label) as between-subjects factors. Contrary to our expectations, we did not find a significant interaction for Time × Number of Standards, *p* = 0.85, η_p_^2^ = 0.000, nor main effects for Time, *p* = 0.39, η_p_^2^ = 0.008, Number of Standards, *p* = 0.98, η_p_^2^ = 0.000, Labeling, *p* = 0.94, η_p_^2^ = 0.001, or interaction effects for Time × Labeling, *p* = 0.45, η_p_^2^ = 0.017, or Time × Labeling × Number of Standards, *p* = 0.99, η_p_^2^ = 0.000. Second, we conducted a similar 2 × 2 × 3 repeated measurement ANOVA with the pretest and posttest scores for the Transfer Explanation Task. This analysis showed a significant main effect of Time, *F*(1,93) = 9.43, *p* < 0.01, η_p_^2^ = 0.09, indicating an improvement from the pretest, *M* = 0.71 (*SD* = 1.18) to the posttest, *M* = 1.05 (*SD* = 1.44). There were no significant main effects for Number of Standards, *p* = 0.52, η_p_^2^ = 0.004, or Labeling, *p* = 0.81, η_p_^2^ = 0.005, nor interaction effects for Time × Number of Standards, *p* = 0.17, η_p_^2^ = 0.02, Time × Labeling, *p* = 0.27, η_p_^2^ = 0.028, or Time × Labeling × Number of Standards, *p* = 0.74, η_p_^2^ = 0.006. That is, the improvement from pretest to posttest in the Explanation Task did not differ between conditions.

### Discussion of Experiment 2

As expected, we found that preschoolers were more likely to induce the concept of material kind when analogical reasoning by comparison was triggered by presenting two objects of the same material rather than only one object. This effect was reflected in the Prediction Task and the Explanation Task, albeit with small effect sizes overall. As in Experiment 1, children’s performance in the baseline assessment, included as a covariate in our statistical models, contributed significantly to children’s performance in the Prediction and Explanation Tasks, with a large effect size. In Experiment 1, we also found that assigning a common label to all objects increased choices of objects of the same material. In contrast to Experiment 1, we used the material labels in a more restricted way in Experiment 2, applying them only to the standards and not to the selection objects. This more restricted use did not improve preschoolers’ performance in the Prediction and Explanation Tasks in comparison to the unlabeled conditions. Our analyses of the Transfer Prediction Task and the Transfer Explanation Task only revealed a small overall gain in the Transfer Explanation Task. Overall, none of the conditions caused specific knowledge transfer effects. However, do our results suggest that the use of labels promotes analogical reasoning by comparison? The lack of a difference between the “two standards/no label” and “two standards/real label” conditions suggests that using material labels has no effect on top of presenting two objects. Given that the instruction and observation of the floating and sinking of two objects might have already invoked the comparison of the two standards, children may not have needed an additional linguistic prompt to align both items.

## General Discussion

### Task Effects of Comparisons of Number of Standards and Linguistic Labels

In our two experiments, we investigated whether triggering analogical reasoning by comparison and additional labeling would enhance preschoolers’ induction of the concept of material kind as a basis for the generation of predictions and explanations in a scientific context for “floating and sinking.” To this end, preschoolers were randomly assigned to conditions that were intended to trigger analogical reasoning by comparison by presenting objects evidenced to sink or float in water (i.e., standards). The objects were presented without labels, with their real material labels, or with made-up labels (Experiment 2 only). The children were then asked to predict which of four selection objects with an unknown status would float or sink and to explain their prediction. As expected, the presentation of two standards rather than one standard supported preschoolers’ induction of the concept of material kind as a basis for generating predictions in both experiments (Ruffman et al., 1993). The benefit of two standards for the provision of explanations only emerged in the second experiment, however. This pattern of findings fits with previous research emphasizing the beneficial role for category and concept learning of triggering analogical reasoning by comparison through the presentation of two standards (Loewenstein et al., 1999; Gentner et al., 2007; Gentner, 2010, 2016). Unlike previous research on preschoolers’ learning through comparison, which employed forced-choice tasks, the present experiments assessed children’s induction of conceptual knowledge for hypothesis generation using tasks that required children to select an object (Prediction Task) and to explain their selection (Explanation Task).

Prior research on analogical reasoning by comparison has provided some evidence that labels as language prompts may be even more beneficial than simply juxtaposing entities or objects. Language is presumed to play a pivotal role because using common labels across different entities may function as an invitation to compare and, as such, to align the similarities and differences of the entities and the encoding and abstraction of a generalizable schema (Gentner and Namy, 1999, 2006; Namy and Gentner, 2002; Gentner, 2010, 2016). In Experiments 1 and 2, we labeled the material comprising each object, potentially providing superordinate category labels. Contrary to our hypothesis, we found an effect of labeling both in the conditions with one standard and with two standards. Since the labels in Experiment 1 were not only applied to the presented standards but also to the four selection objects, it is likely that the children’s prior conceptual knowledge of material kind was activated in the condition with a single standard, facilitating responses based on the same material. The use of real material labels in Experiment 1 differed from the approach of Namy and Gentner (2002), who used made-up labels. In Experiment 1, at least some of the children were familiar with material labels from everyday life contexts, as indicated by their performance in the pretest, in which the children were asked to label the material of various objects. In Experiment 2, we therefore used labels in a more restricted way in order to differentiate between the effects of prior conceptual knowledge and the facilitation of comparison by labeling. We found no effect for labeling with this more restricted use. Children’s predictions and explanations did not differ from the conditions without labels, neither when made-up labels were used nor when real labels were used. In Gentner and Namy’s (1999) Experiment 2, a label and a no-label condition were contrasted, as well as a compare (two standards) and a non-compare (one standard) condition. Gentner and Namy found that the label/two-standard standard condition significantly increased appropriate responses not only in contrast to both single standard conditions but also to the unlabeled/two-standard condition. How may these differences be explained? We suppose that even our “two standards without labeling” condition sufficed as an invitation for analogical reasoning by comparison because the children could also observe whether these objects floated or sank as empirical evidence associated with the respective objects. These observations may have created an alignable similarity between the two objects, helping the preschoolers to induce the concept of material kind, in both Experiment 1 and Experiment 2. By contrast, Namy and Gentner only showed pictures of one versus two objects; thus, verbal support may have been more important than in our experiments. Moreover, in our experiments, all the standard objects of the same material showed the same floating/sinking behavior. That is, the covariation between objects and observed object behavior was perfect. In this regard, our findings fit with previous research indicating that even 4-year-olds can coordinate theory and evidence if there is a perfect covariation (Tullos and Woolley, 2009).

In Experiment 2, preschoolers’ performance in the conceptual knowledge transfer test did not differ between conditions. Even so, the overall significant gains from pretest to posttest on the dependent measure of explanations may provide a first, albeit weak indication that a process of conceptual restructuring was initiated. Because conceptual restructuring is a process in which naïve conceptions are gradually extended and refined (Vosniadou et al., 2001), this process needs to be continued and deepened since children typically need additional opportunities to overcome their misconceptions (Leuchter et al., 2014). The slow process of conceptual restructuring may explain why the benefits of task environments inviting analogical reasoning by comparison are rather small in our experiments. Our tasks required preschoolers to overcome their misconceptions and construct novel conceptual knowledge in a science domain with a high degree of prior knowledge—the concept of material kind within the context of floating and sinking. Developing such knowledge may be more challenging than the basic-category learning studied in previous research on the benefits of comparison (e.g., Namy and Gentner, 2002).

### Limitations

Throughout this paper, we suggested that analogical reasoning by comparison benefits young children’s generation of predictions and explanations as a central aspect of scientific reasoning. The cognitive models of analogical reasoning by comparison provide conceptualizations of how children actually engage in this process: When learners engage in analogical reasoning by comparison, they identify similarities and differences between entities, they may align them and abstract a schema, they may re-represent their existing conceptions, and they may project inferences based on the results of these processes (Gentner, 2010). In our experiments, we investigated only the induction of one specific basic science concept: the concept of material kind. We did not investigate hypothesis generation in other science domains that vary in children’s prior conceptual knowledge and that may have an impact on children’s generation of predictions and explanations. Therefore, further research is needed to investigate the induction of other scientific concepts so that the claim that analogical reasoning by comparison has benefits for scientific reasoning in general may be strengthened. Moreover, while we did assess children’s prior knowledge of material kind, we did not look at individual differences and their influences on the process of hypothesis generation. Thus, further research should address the interaction of prior knowledge and the generation of predictions and explanations. Generally, while hypothesis generation is a central aspect of scientific reasoning, it will be important for future research to investigate whether other elements of the scientific inquiry cycle, such as the generation of experiments, the interpretation of data patterns, and the evaluation of evidence, would also benefit from interventions aimed to trigger analogical reasoning by comparison.

While both our studies were conducted with preschoolers, we included a wide age range of children, between 4 and 7 years. Additional exploratory analyses indicated that there were no significant differences between different age groups. However, our study design does not make it possible to fully disentangle the possible impact of age on the tasks used in the present experiments. On the basis of results summarized by Gentner (2010) and Hespos et al. (2020), we expect analogical reasoning by comparison to be a cognitive mechanism available to children starting at an early age. However, further research is needed to investigate age differences with the task formats that we employed in our experiments. For example, it is possible that age may be a more important factor in the Explanation Task, which required children to produce verbal answers, than in the Prediction Task, even though we did not find effects from age in either task format. Here, we have to acknowledge that the power of the present experiments is not sufficient to detect such differences (i.e., interactions between age, tasks, and conditions). A closer look at age differences may also illuminate the differences in performance between the dependent measures of Prediction and Explanation Tasks found in Experiments 1 and 2. Therefore, task designs taking into account individual differences in children’s responses could also contribute to understanding the contingencies between children’s predictions and explanations.

### Conclusion

In our two experiments, we investigated whether triggering analogical reasoning by comparison would benefit children’s predictions and explanations of objects’ floating or sinking based on the concept of material kind. On the one hand, we triggered analogical reasoning by comparison by presenting two objects of the same material simultaneously and found some evidence that this indeed benefited children’s induction of the concept of material kind for hypothesis generation. On the other hand, unexpectedly, the additional provision of language prompts did not increase the effect of presenting two objects. In previous research, such additional support was often necessary for concept learning, especially with young children. It may be that our task structure provided a different kind of support for triggering comparison because our tasks included the demonstration of the floating and sinking behavior of the standards. This speculation may provide interesting directions for future research. Our experiments therefore only give a first hint that analogical reasoning by comparison may be helpful for the induction of science concepts in a scientific reasoning context.

## Data Availability Statement

The datasets generated for this study are available on request to the corresponding author.

## Ethics Statement

Ethical review and approval was not required for the study on human participants in accordance with the local legislation and institutional requirements. Written informed consent to participate in this study was provided by the participants’ legal guardian/next of kin.

## Author Contributions

HS and IH contributed the conception and design of the experiments. ML, HS, IH, and LS conducted the Experiments 1 and 2 in Germany and Switzerland. HS and IH performed the statistical analyses. ML, HS, and IH wrote a first draft of the manuscript. All the authors contributed to manuscript revision and approved the submitted version.

## Conflict of Interest

The authors declare that the research was conducted in the absence of any commercial or financial relationships that could be construed as a potential conflict of interest.

## References

[B1] Alfieri L. Nokes-Malach T. J. Schunn C. D. (2013). Learning through case comparisons: a meta-analytic review. *Educ. Psychol.* 48 87–113. 10.1080/00461520.2013.775712

[B2] Arunachalam S. Waxman S. R. (2010). Language and conceptual development. *Wiley Interdiscip. Rev. Cogn. Sci.* 1 548–558. 10.1002/wcs.37 26271502

[B3] Carey S. (2000). Science education as conceptual change. *J. Appl. Dev. Psychol.* 21 13–19.

[B4] Childers J. (2020). *Language and Content Acquisition from Infancy through Childhood: Learning from Multiple Exemplars.* Berlin: Springer.

[B5] Chinn C. A. Brewer W. F. (2001). Models of data: a theory of how people evaluate data. *Cogn. Instr.* 19 323–393. 10.1207/S1532690XCI1903_3

[B6] Chinn C. A. Malhotra B. A. (2002). Children’s responses to anomalous scientific data: how is conceptual change impeded? *J. Educ. Psychol.* 94 327 10.1037//0022-0663.94.2.327

[B7] Christie S. (2020). “Multiple exemplars of relations,” in *Language and Content Acquisition from Infancy through Childhood: Learning from Multiple Exemplars*, ed. Childers J. (Berlin: Springer), 221–246. 10.1007/978-3-030-35594-4_11

[B8] Christie S. Gentner D. (2010). Where hypotheses come from: learning new relations by structural alignment. *J. Cogn. Dev.* 11 356–373. 10.1080/15248371003700015

[B9] Davidson N. S. Gelman S. A. (1990). Inductions from novel categories: the role of language and conceptual structure. *Cog. Dev.* 5 151–176. 10.1016/0885-2014(90)90024-n

[B10] Dickinson D. K. (1987). The development of a concept of material kind. *Sci. Educ.* 71 615–628. 10.1002/sce.3730710412

[B11] Driver R. Newton P. Osborne J. (2009). Establishing the norms of scientific argumentation in classrooms. *Sci. Educ.* 84 1–312. 10.1007/s10503-017-9424-z

[B12] Dunbar K. Klahr D. (2012). “Scientific thinking and reasoning,” in *The Oxford Handbook of Thinking and Reasoning*, eds Holyoak K. J. Morrison R. G. (Oxford: Oxford University Press), 701–718.

[B13] Gelman S. A. Collman P. Maccoby E. E. (1986). Inferring properties from categories versus inferring categories from properties: the case of gender. *Child Dev.* 57 396–404. 10.2307/1130595

[B14] Gelman S. A. Markman E. M. (1986). Categories and induction in young children. *Cognition* 23 183–209. 10.1016/0010-0277(86)90034-x 3791915

[B15] Gentner D. (2010). Bootstrapping the mind: analogical processes and symbol systems. *Cogn. Sci.* 34 752–775. 10.1111/j.1551-6709.2010.01114.x 21564235

[B16] Gentner D. (2016). Language as cognitive tool kit: how language supports relational thought. *Am. Psychol.* 71 650–657. 10.1037/amp0000082 27977235

[B17] Gentner D. Hoyos C. (2017). Analogy and abstraction. *Top. Cogn. Sci.* 9 672–693. 10.1111/tops.12278 28621480

[B18] Gentner D. Loewenstein J. Hung B. (2007). Comparison facilitates children’s learning of names for parts. *J. Cogn. Dev.* 8 285–307. 10.1080/15248370701446434

[B19] Gentner D. Medina J. (1998). Similarity and the development of rules. *Cognition* 65 263–297. 10.1016/s0010-0277(98)00002-x 9557385

[B20] Gentner D. Namy L. L. (1999). Comparison in the development of categories. *Cog. Dev.* 14 487–513. 10.1016/s0885-2014(99)00016-7

[B21] Gentner D. Namy L. L. (2006). Analogical processes in language learning. *Curr. Dir. Psychol. Sci.* 15 297–301. 10.1111/j.1467-8721.2006.00456.x

[B22] Gentner D. Rattermann M. (1991). “Language and the career of similarity,” in *Perspectives on Language and Thought: Interrelations in Development*, eds Gelman S. Byrnes J. (Cambridge: Cambridge University Press), 225–277. 10.1017/cbo9780511983689.008

[B23] Gentner D. Smith L. (2012). “Analogical reasoning,” in *Encyclopedia of Human Behavior* 2nd Ed V. S. Ramachandran (Oxford: Elsevier), 130–136.

[B24] Graham S. A. Booth A. E. Waxman S. R. (2012). Words are not merely features: only consistently applied nouns guide 4-year-olds’ inferences about object categories. *Lang. Learn. Dev.* 8 136–145. 10.1080/15475441.2011.599304

[B25] Gropen J. Kook J. F. Hoisington C. Clark-Chiarelli N. (2017). Foundations of science literacy: efficacy of a preschool professional development program in science on classroom instruction, teachers’ pedagogical content knowledge, and children’s observations and predictions. *Early Educ. Dev.* 28 607–631. 10.1080/10409289.2017.1279527

[B26] Hardy I. Jonen A. Möller K. Stern E. (2006). Effects of instructional support within constructivist learning environments for elementary school students’ understanding of “floating and sinking.”. *J. Educ. Psychol.* 98 307–326. 10.1037/0022-0663.98.2.30

[B27] Hespos S. Anderson E. Gentner D. (2020). “Structure mapping processes enable infants’ learning across domains including language,” in *Language and Content Acquisition from Infancy through Childhood: Learning from Multiple Exemplars*, ed. Childers J. (Berlin: Springer), 97–104. 10.1080/01690960344000143

[B28] Holland J. H. Holyoak K. J. Nisbett R. E. Thagard P. R. (1989). *Induction: Processes of Inference, Learning, and Discovery.* Cambridge: MIT Press.

[B29] Holyoak K. J. (2005). “Analogy,” in *The Cambridge Handbook of Thinking and Reasoning*, eds Holyoak K. J. Morrison R. G. (Cambridge: Cambridge University Press), 117–142.

[B30] Johanson M. Papafragou A. (2016). The influence of labels and facts on children’s and adults’ categorization. *J. Exp. Child Psychol.* 144 130–151. 10.1016/j.jecp.2015.11.010 26735976

[B31] Klahr D. (2000). *Exploring Science: The Cognition and Development of Discovery Processes.* Cambridge, MA: MIT Press.

[B32] Klahr D. Dunbar K. (1988). Dual space search during scientific reasoning. *Cogn. Sci.* 12 1–48. 10.1207/s15516709cog1201_1

[B33] Koerber S. Sodian B. Thoermer C. Nett U. (2005). Scientific reasoning in young children: preschoolers’ ability to evaluate covariation evidence. *Swiss J. Psychol.* 64 141–152. 10.1024/1421-0185.64.3.141

[B34] Köksal-Tuncer Ö Sodian B. (2018). The development of scientific reasoning: hypothesis testing and argumentation from evidence in young children. *Cog. Dev.* 48 135–145. 10.1016/j.cogdev.2018.06.011

[B35] Kuhn D. (2010). “What is scientific thinking and how does it develop?,” in *Handbook of Childhood Cognitive Development*, 2nd Edn ed. Goswami U. (Oxford: Blackwell), 472–534.

[B36] Kurtz K. J. Loewenstein J. (2007). Converging on a new role for analogy in problem solving and retrieval: when two problems are better than one. *Mem. Cognit.* 35 334–341. 10.3758/bf03193454 17645174

[B37] Kurtz K. J. Miao C. H. Gentner D. (2001). Learning by analogical bootstrapping. *J. Learn. Sci.* 10 417–446. 10.1207/s15327809jls1004new_2

[B38] Leuchter M. Saalbach H. Hardy I. (2014). Designing science learning in the first years of schooling: an intervention study with sequenced learning material on the topic of “floating and sinking.”. *Int. J. Sci. Educ.* 36 1751–1771. 10.1080/09500693.2013.878482

[B39] Loewenstein J. Gentner D. (2001). Spatial mapping in preschoolers: close comparisons facilitate far mappings. *J. Cogn. Dev.* 2 189–219. 10.1207/s15327647jcd0202_4

[B40] Loewenstein J. Thompson L. Gentner D. (1999). Analogical encoding facilitates knowledge transfer in negotiation. *Psychon. Bull. Rev.* 6 586–559. 10.3758/bf03212967 10682201

[B41] Mercier H. (2011). Reasoning serves argumentation in children. *Cog. Dev.* 26 177–191. 10.1016/j.cogdev.2010.12.001

[B42] Mercier H. Sperber D. (2009). “Intuitive and reflective inferences,” in *Two Minds: Dual Processes and Beyond*, eds Evans J. Frankish K. (Oxford: Oxford University Press), 149–170. 10.1093/acprof:oso/9780199230167.003.0007

[B43] Morris B. J. Masnick A. M. Zimmerman C. Croker S. (2012). “The emergence of scientific reasoning,” in *Current Topics in Children’s Learning and Cognition*, eds Kloos H. Morris B. Amaral J. (London: IntechOpen), 61–82. 10.5772/53885

[B44] Namy L. L. Gentner D. (2002). Making a silk purse out of two sow’s ears: young children’s use of comparison in category learning. *J. Exp. Psychol. Gen.* 131 5–15. 10.1037//O096-LWS.131.I.5 11900103

[B45] Nguyen S. P. Gelman A. (2012). Generic language facilitates children’s cross-classification. *Cog. Dev.* 27 154–167. 10.1016/j.cogdev.2012.01.001 22888182 PMC3414382

[B46] Penner D. Klahr D. (1996). The interaction of domain-specific knowledge and domain general discovery strategies: a study with sinking objects. *Child Dev.* 67 2709–2727. 10.2307/1131748 9071759

[B47] Piekny J. Grube D. Maehler C. (2014). The development of experimentation and evidence evaluation skills at preschool age. *Int. J. Sci. Educ.* 36 334–354. 10.1080/09500693.2013.776192

[B48] Piekny J. Maehler C. (2013). Scientific reasoning in early and middle childhood: the development of domain-general evidence evaluation, experimentation, and hypothesis generation skills. *Br. J. Dev. Psychol.* 31 153–179. 10.1111/j.2044835X.2012.02082.x 23659889

[B49] Ruffman T. Perner J. Olson D. R. Doherty M. (1993). Reflecting on scientific thinking: children’s understanding of the hypothesis–evidence relation. *Child Dev.* 64 1617–1636. 10.2307/1131459 8112110

[B50] Saalbach H. Imai M. Schalk L. (2012). Grammatical gender and inferences about biological properties in german-speaking children. *Cogn. Sci.* 36 1251–1267. 10.1111/j.1551-6709.2012.01251.x 22578067

[B51] Sandoval W. Sodian B. Koerber S. Wong J. (2014). Developing children’s early competencies to engage with science. *Educ. Psychol.* 49 139–152. 10.1080/00461520.2014.917589

[B52] Schalk L. Saalbach H. Stern E. (2016). Approaches to foster transfer of formal principles: which route to take? *PLoS One* 11:e0148787. 10.1371/journal.pone.0148787 26871902 PMC4752471

[B53] Schneider M. Vamvakoussi X. van Dooren W. (2012). “Conceptual change,” in *Encyclopedia of the Sciences of Learning*, ed. Seel N. (New York: Springer), 735–738.

[B54] Schulz L. E. Gopnik A. Glymour C. (2007). Preschool children learn about causal structure from conditional interventions. *Dev. Sci.* 10 322–332. 10.1111/j.1467-7687.2007.00587.x 17444973

[B55] Smith C. Carey S. Wiser M. (1985). On differentiation: a case study of the development of the concepts of size, weight, and density. *Cognition* 21 177–237. 10.1016/0010-0277(85)90025-3 3830547

[B56] Sodian B. Zaitchik D. Carey S. (1991). Young children’s differentiation of hypothetical beliefs from evidence. *Child Dev.* 62 753–766. 10.2307/1131175

[B57] Tullos A. Woolley J. (2009). The development of children’s ability to use evidence to infer reality status. *Child Dev.* 80 101–114. 10.1111/j.1467-8624.2008.01248.x 19236395 PMC2743968

[B58] van der Graaf J. Segers E. Verhoeven L. (2016). Scientific reasoning in kindergarten: cognitive factors in experimentation and evidence evaluation. *Learn. Individ. Differ.* 49 190–200. 10.1016/j.lindif.2016.06.006

[B59] Vosniadou S. Ioannides C. Dimitrakopoulou A. Papademetriou E. (2001). Designing learning environments to promote conceptual change in science. *Learn. Instr.* 11 381–419. 10.1016/S0959-4752(00)00038-4

[B60] Zimmerman C. (2007). The development of scientific thinking skills in elementary and middle school. *Dev. Rev.* 27 172–223. 10.1016/j.dr.2006.12.001

